# Non-Invasive Monitoring and Differentiation of Aging Mice Treated with Goat Whey Powder by an Electronic Nose Coupled with Chemometric Methods †

**DOI:** 10.3390/s25051496

**Published:** 2025-02-28

**Authors:** Guilong Zhu, Yahe Yang, Fumei Zhang, Jia Wei, Xiaojing Tian, Lixia Liu, Zuolin Ma, Guoheng Zhang

**Affiliations:** 1 School of Life Sciences and Engineering, Northwest Minzu University, Lanzhou 730124, China; zgl981112@outlook.com (G.Z.); weijiapku@xbmu.edu.cn (J.W.); 2 Gansu Engineering Research Center of Ecological Environment Intelligent Networking, College of Electrical Engineering, Northwest Minzu University, Lanzhou 730030, China; yahe.yang@network.rca.ac.uk (Y.Y.); dqzgh@xbmu.edu.cn (G.Z.); 3 Gansu Tech Innovation Center of Animal Cell, Biomedical Research Center, Northwest Minzu University, Lanzhou 730030, China; 278061349@xbmu.edu.cn (F.Z.); m.zuolin@gmail.com (Z.M.)

**Keywords:** electronic nose, goat whey powder, canonical discriminant analysis, canonical correlation analysis, aging mice model

## Abstract

For the evaluation of food efficacy, in vitro experiments and cell and animal models are heavily relied on, with a need for quick and non-invasive monitoring methods. In this study, the fecal odor of aging mice supplemented with goat whey powder was obtained by an E-nose, and the correlation between odor information and the antioxidant indexes, serum antibody, cytokine, and intestinal bacteria were analyzed, aiming to establish a non-invasive method for monitoring and differentiating the effect of goat whey powder. As a result, the fecal odor differed with intervention groups and intervention time, and most of the sensor responses were significantly correlated with weight gain rate, SOD activity, and MDA content. For serum antibodies, cytokines, IL-2, and IL-6 were negatively correlated with the responses of sensor S7. A strong correlation was found between the E-nose sensor responses and the dominant intestinal bacteria. The E-nose could differentiate aging mice of different intervention times and intervention groups with canonical discriminate analysis (CDA). The effective predictive model was built by multiple linear regression (MLR) and a multilayer perceptron neural network (MLP) for SOD, MDA, and weight gain rate, with R^2^ ranging from 0.1571 to 0.6361. These results indicated that E-nose technology could be used in the tracking of goat whey powder intervention in aging mice.

## 1. Introduction

Whey powder is a natural by-product in cheese production, which contains a variety of protein components, such as casein, *β*-lactoglobulin (*β*-LG), *α*-Lactalbumin (*α*-La), immunoglobulin, lactoferrin (*Lf*), enzymes, and low-molecular-degradation products [1,2]. As a source of diverse biologically active compounds with the benefits of promoting powder synthesis [3], mineral absorption, antibacterial [4], anticancer, and antioxidant effects [5,6,7,8], whey powder is used in the food industry and clinical treatment for maintaining and improving immunity, resisting free radicals, delaying the aging process, improving renal function, and promoting wound healing [9]. Higher in alpha-lactalbumin, beta-lactoglobulin, and serum albumin [10], goat whey powder exhibited good effects in beneficially altering the intestinal microbiota, improving memory and anxiolytic-like behavior in rats during aging [11], and in anti-aging [12].

For the antioxidant efficacy evaluation of whey powder and dietary intervention [13], cell models, animal model experiments, and human clinical trials are heavily relied upon. However, the experimental animals need to be killed to obtain serum or livers for the detection of physiological, biochemical, and morphological indicators. And the analysis processes of these indications are complicated in terms of operation, high in terms of cost, and time-consuming. Moreover, the anti-antioxidant efficacy evaluation needs to be conducted in a comprehensive manner, comprising lipid oxidation products, protein oxidation products, antioxidant enzyme activity, and antioxidant substance-reduced glutathione. More importantly, the needs of experimental animals resulted in an increasing trend of its usage year over year [14]. It is important to explore quick and non-invasive methods to evaluate the effect of animal experiments.

Previous reports showed that the composition and content of metabolites [15,16] changed with the intestinal microflora structure, which reflects the performance of different diets and nutrition regulation [17]. And the metabolic products accumulate in the exhaled breath, urine, sweat, blood, and feces, characterized by odor change, which can be traced by electronic nose (E-nose) sensors [18,19]. As a device that simulates the mammalian olfactory system, the E-nose is sensitive to volatiles released by the sample. With the advantages of being non-invasive and fast, the E-nose has been applied in various fields such as food processing and quality detection, pest detection, environmental pollutant and odor monitoring, drug development, medical diagnosis, and monitoring. Fecal odor information is an option for the non-invasive evaluation of functional properties. Using feces samples, the influence of external conditions, such as gender and age [20], body mass index (BMI), smoking history, diet, and enteral feeding composition [21,22], comorbidity and medication, sampling conditions, and environmental factors [23] were traced by the response signals of the E-nose [24]. For in vivo evaluation of functional components, with the odor information of volatile metabolites in feces obtained by E-nose, the odor response of the litchi shell proanthocyanidin oligomers by the gavage of SD rats was significantly lower than that of the blank group samples [25]. The heat stress and modulating dietary cinnamon effects in the pig model were studied with a gas sensor capsule, finding that the gastric gas profile provided a better understanding of the physiological changes [26]. Based on the odor information of feces at three observation points, a significant difference in the odor of fecal volatile components was found between breast milk (BM) and formula milk (FM) feeding methods [21]. The microbial enterotype could be reliably predicted using the decision tree model based on the stool samples’ odor collected by two sensors [22]. And the blood pressure changes in spontaneously hypertensive rats were tracked and predicted using fecal odor information [27]. A non-invasive evaluation method has been established for the antioxidant properties of bovine whey protein using fecal odor in our previous study. Additionally, the changes in fecal odor during the process of D-galactose-induced aging in mice fed with bovine whey powder were tracked by an E-nose, revealing a strong correlation between fecal odor information and the antioxidant activity indicators, SOD, and MDA [28].

For the prediction of intestinal flora structure, a prediction model for the flora data was constructed based on the odor fingerprint of intestinal fermentation broth samples [29]. Differences in intervention effects of different doses of bovine whey protein were distinguished with PCA and CDA using E-nose responses, and the prediction model for body weight was established by multiple linear regression analysis (MLR) with R^2^ = 0.1227. The E-nose proved to be useful in the differentiation of odor-monitoring changes in the animal model [28].

Our previous studies have shown that for aging mice, intervention with different types of whey protein significantly increased the antioxidant capacity and the abundance of lactobacilli [12,27] while inhibiting the abundance of mycoplasma. In this study, we tried to find the relation between odor information and various physiological indexes, including serum antibodies and cytokines, and the intestinal bacteria of treated mice and to build a fast, non-invasive method for evaluating the effects of goat whey powder on aging mice.

## 2. Materials and Methods

### 2.1. Aging Animal Model

**Figure 1:**
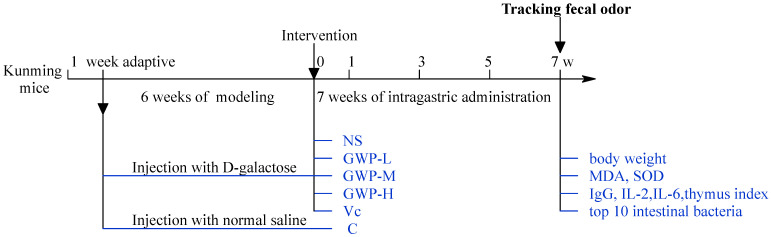
Experiment design. C: non-treatment control; NS: negative control; Vc: positive control; GWP-L, GWP-M, and GWP-H represent the low-, middle-, and high-concentration GWP intervention groups, respectively.

After 1 week of acclimatization, the aging mice were induced by subcutaneous injection with 10% D-galactose dissolved in normal saline (0.25 mL/20 g/day) for 6 weeks. And different doses of (low: 100 mg/kg, mid: 200 mg/kg, high: 300 mg/kg) goat whey powder (GWP) were given to the aging models for 7 weeks (intragastric administration). The processing details are described in our former article and in Figure 1. All animals used were reviewed and approved by Northwest Minzu University with the ethical review number xbmu-sm-201902.

### 2.2. Test Methodology

#### 2.2.1. Weight, Antioxidant Ability, Serum Antibody, and Cytokine Determination

The preparation of goat whey powder; intervention in aging mice; and the corresponding results of body weight, antioxidant ability (MDA content, serum SOD activity), serum antibodies, and cytokines (IgG, thymus index, IL-2, IL-6) were described in formal article [12].

#### 2.2.2. Intestinal Bacteria

The procedure for intestinal microbial DNA isolation, 16sRNA sequencing, and the results of top 10 intestinal bacteria were described in earlier article [12].

#### 2.2.3. Collection of Fecal Samples

Forty pellets of female mice feces were collected at the beginning of intervention and at the 1st, 3rd, 5th, and 7th weeks of intervention. The collected pellets were placed in sterile centrifuge tube of 5 mL and stored at −80 °C.

#### 2.2.4. Electronic Nose (E-Nose) and the Detection Procedure

To track the odor of fecal samples of mice who had interventions via goat whey powder, a PEN3 E-nose (Airsense Corporation, Schwerin, Germany) was used. The sensor array of E-nose system was composed of 10 metal oxide sensors. The sensors used and their main applications are described in Table 1. The software Win Muster v.1.6 was used for data recording.

Before being detected by E-nose, the fecal samples were brought to room temperature. The headspace used for the detection of E-nose was generated by the following processes. Without any preparation, 1 drop of fecal sample was placed in a beaker of 150 mL, sealed by plastic, and kept for 10 min at room temperature of 25 °C ± 3 °C for the headspace to stabilize. During sampling, the stabilized headspace was transferred into the sensor chamber at a flow rate of 200 mL min^−1^. The detection procedure was as follows: for each sample, the measurement time was set to 60 s at an interval of 1 s, and the sensors were rinsed for 80 s with clean air before the detection of next sample [28]. When the headspace gas is transferred into the sensor chamber, the volatiles are adsorbed by the sensor and generate response signals, which are sent to the signal processing subsystem, processed, and output sensor responses are given as G/G_0_, where G and G_0_ express the resistance of a sensor in clean air and in detecting gas, respectively. All the samples were detected at room temperature with 40 duplications.

### 2.3. Statistical Analysis

The ability of E-nose in the assessment of goat whey powder on aging mice was studied with the help of chemometric method. Duncan’s test was conducted to study the difference in sensor response for different groups. Canonical correlation analysis (CCA) was used to study the correlation between odor information of E-nose and physiological indexes, serum antibodies and cytokines, and intestinal bacteria of treated mice. Canonical discriminate analysis (CDA) was used to monitor the odor change of fecal samples of mice affected by goat whey and differentiate the different intervention times and groups. To build the predictive model for weight gain rate, multiple linear regression (MLR) and multilayer perceptron neural network (MLP) were used. For data analysis, the SAS version 8 (SAS Institute Inc., Gary, IN, USA) was used. The figures were plotted with Origin Pro 8.

## 3. Results and Discussion

### 3.1. Typical Response of E-Nose Sensor to Fecal Samples

**Figure 2:**
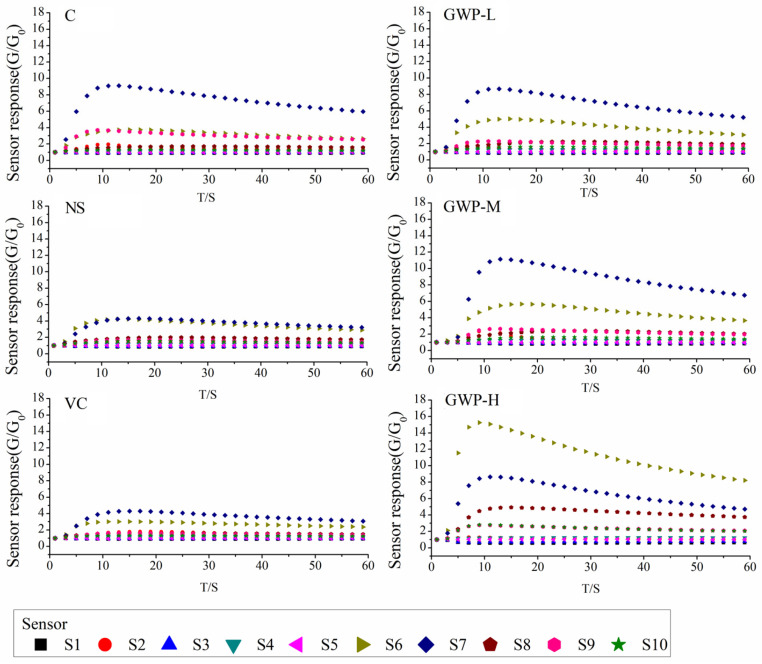
Sensor response curves of fecal samples of aging mice with different interventions (7th week).

The typical responses of E-nose sensors to fecal samples of aging mice with different interventions in the 7th week are shown in Figure 2. The sensor responded quickly to the volatiles in fecal samples, then reached the maximum value at around the 10th s and stabilized after 50 s of collection time. For samples treated with different intervention doses and time, the tendency and intensity of the sensor responses were quite different, especially for sensors S6, S7, S8, and S9. The stabilized sensor responses at the 60th s were extracted and used for further analysis.

The analysis of variance for the test groups was carried out with the Duncan test, as shown in Table 2. Compared with the C group, the fecal odors of aging mice in the NS group were lower in the responses of sensors S1–S5, S7, and S9 and higher in the responses of sensors S6, S8, and S10. After a vitamin C intervention, the aging mice recovered to a similar state as the blank mice in the C group, with no difference in the response of sensors S1, S3, S5, S6, S8, and S10. The signals of sensors S2, S4, and S7 were improved, although significantly different than that of the C group. Goat whey protein-treated aging mice showed similar values of sensor responses with that of the Vc group or were even better, especially for low and medium doses of goat whey protein, while sensor responses to high doses of goat whey protein were close to that of the NS group.

### 3.2. Correlation Between Sensor Responses and Physiological Indexes

**Figure 3:**
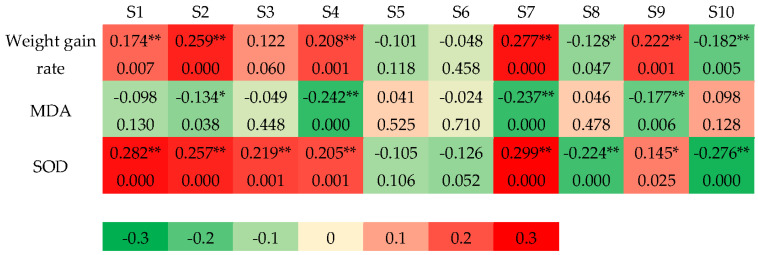
CCA results of E-nose sensor responses and antioxidant indexes (the 7th week). Note: Colors close to 0 and 1/−1 correspond to the low and high correlations of sensor signals, respectively. The values in the cell represent the correlation coefficient (upper) and *p* value (lower). (*) *p* < 0.05, (**) *p* < 0.01.

**Figure 4:**
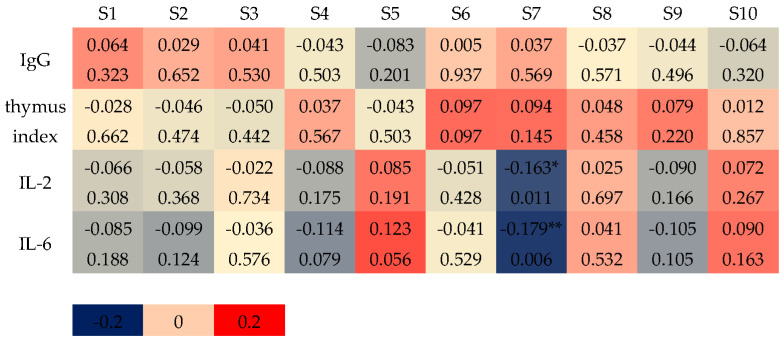
CCA results of E-nose sensor responses and immune index (the 7th week). Note: Colors close to 0 and 1/−1 correspond to the low and high correlations of sensor signals, respectively. The values in the cell represent the correlation coefficient (upper) and *p* value (lower). (*) *p* < 0.05, (**) *p* < 0.01.

**Figure 5:**
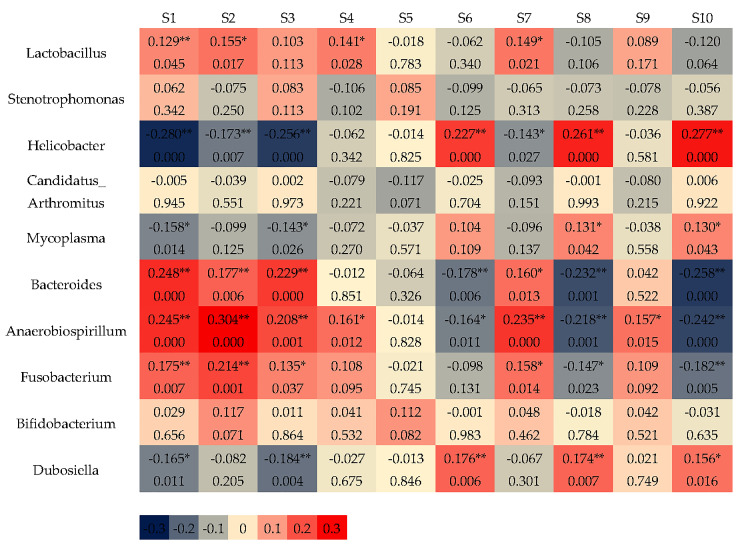
CCA results of E-nose sensor responses and intestinal flora (the 7th week). Note: Colors close to 0 and 1/−1 correspond to the low and high correlations of sensor signals, respectively. The values in the cell represent the correlation coefficient (upper) and *p* value (lower). (*) *p* < 0.05, (**) *p* < 0.01.

To study the possibility of the E-nose in the assessment of goat whey protein on aging mice, the correlation between odor information of the E-nose and physiological indexes, serum antibodies and cytokines, and intestinal bacteria of treated mice were analyzed by Canonical correlation analysis (CCA), and the results were shown in Figure 3, Figure 4 and Figure 5.

#### 3.2.1. Correlation Between Sensor Responses and Antioxidant Indexes

As shown in Figure 3, it was found by CCA that the weight gain rate was positively correlated with the responses of sensors S1, S2, S4, S7, and S9 (*p* < 0.01) and negatively correlated with the responses of sensors S8 and S10 at *p* < 0.01. MDA content was negatively correlated with the responses of sensors S4, S7, and S9 at *p* < 0.01 and S2 at *p* < 0.05. SOD activity was positively correlated with the responses of sensors S1, S2, S3, S4, and S7 at *p* < 0.01 and S9 at *p* < 0.05 and negatively correlated with the responses of sensors S8 and S10 at *p* < 0.01.

The antioxidant indicators of weight gain rate, MDA content, and serum SOD activity were significantly correlated with E-nose responses.

#### 3.2.2. Correlation Between Sensor Responses and Serum Antibodies and Cytokines

As shown in Figure 4, using CCA, it was found that IL-2 and IL-6 were negatively correlated with the responses of sensor S7, while the correlations between the thymus index, IgG, and sensor responses were not significant.

The correlation between the odor information of the E-nose and the immune indexes of mice was weaker, so it is difficult to reflect the immune performance of goat whey powder using the odor information of the fecal E-nose.

#### 3.2.3. Correlation Between Sensor Responses and Intestinal Bacteria

The correlation between gut intestinal bacteria (top 10 relative abundance) and E-nose responses is shown in Figure 5. It was found that *Lactobacillus* was positively correlated with the responses of sensors S1 (*p* < 0.01), S2, S4, and S7 (*p* < 0.05). *Helicobacter* was positively correlated with the responses of sensors S6, S8, and S10 (*p* < 0.01) and negatively correlated with the responses of sensors S1, S2, and S3 at *p* < 0.01 and S7 at *p* < 0.05. *Mycoplasma* was positively correlated with the responses of sensors S8 and S10 (*p* < 0.05) and negatively correlated with the responses of sensors S1 and S3 at *p* < 0.05. *Bacteroides* were positively correlated with the responses of sensors S1, S2, S3 (*p* < 0.01), and S7 (*p* < 0.05) and negatively correlated with the responses of sensors S6, S8, and S10 (*p* < 0.01). *Anaerobiospirillum* was positively correlated with the responses of sensors S1, S2, S3, and S7 at *p* < 0.01 and S4 and S9 at *p* < 0.05 and negatively correlated with the responses of sensors S6 (*p* < 0.05), S8, and S10 (*p* < 0.01). *Fusobacterium* was positively correlated with the responses of sensors S1 and S2 (*p* < 0.01) and S3 and S7 (*p* < 0.05) and negatively correlated with the responses of sensors S8 (*p* < 0.05) and S10 (*p* < 0.01). *Dubosiella* was positively correlated with the responses of sensors S6 and S8 (*p* < 0.01) and S10 (*p* < 0.05) and negatively correlated with the responses of sensors S1 (*p* < 0.05) and S3 (*p* < 0.01). Leaving *Stenotrophomonas*, *Candidatus_Arthromitus* and *Bifidobacterium* were not correlated with the responses of the E-nose sensor.

The E-nose sensor had a strong correlation with the dominant intestinal bacteria.

### 3.3. Monitoring the GWP Intervention Process by E-Nose

**Figure 6:**
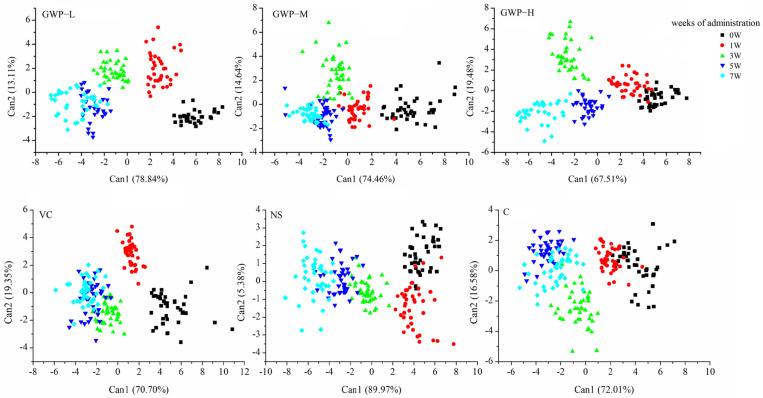
CDA scatter plot of the fecal samples of aging mice for different interventions and time based on E-nose responses. C: non-treatment control; NS: negative control; Vc: positive control; GWP-L, GWP-M, and GWP-H represent the low-, middle-, and high-concentration GWP intervention groups, respectively.

**Figure 7:**
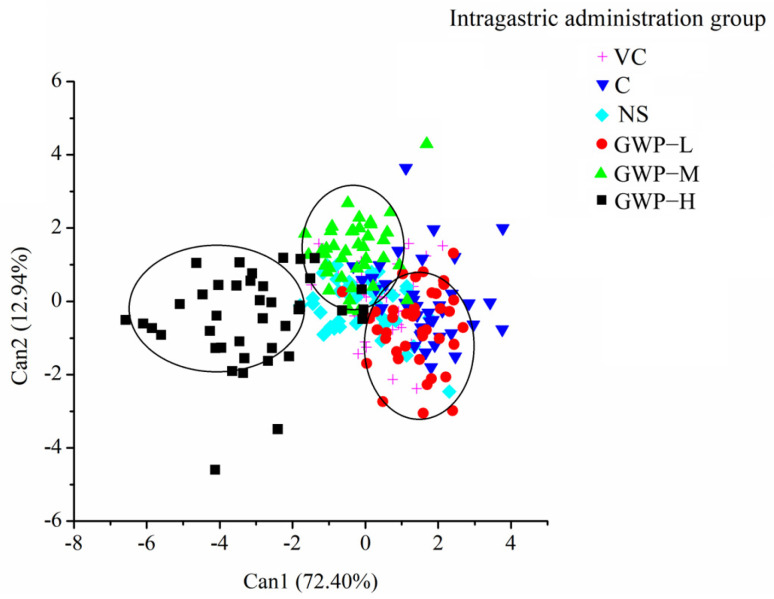
CDA scatter plot of fecal samples of aging mice intervened for 7 weeks based on E-nose responses.

The odor of fecal samples of aging mice changed due to the interventions and time, which were detected by the E-nose. To differentiate the changes between interventions and time, the sensor responses of the E-nose at the 60th s were extracted and analyzed by CDA, and the results are shown in Figure 6 and Figure 7.

For all the treatments, the odor of fecal samples changed during 7 weeks of interventions and differed with each other. These changes were observed in CDA results. As shown in Figure 6, the first two cans explained 91.95%, 89.10%, 86.99%, 90.05%, 95.35%, and 88.59% of the total variance, providing most of the odor information of fecal samples. The scatters of different intervention weeks are located along with the decrease in Can1. The fecal samples collected at the early stage of intervention in the 0th and 1st weeks could be differentiated from the latter weeks. For samples collected in the 5th and 7th weeks, they overlapped with each other, indicating small changes were occurring. Although there were some scatters that overlapped with the adjacent groups, the intervention weeks of different treatments for the fecal samples of aging mice could be differentiated based on the odor information of the fecal samples.

After 7 weeks of intervention, the effect of goat whey powder on aging mice was compared with that of the other groups. The CDA results are shown in Figure 7. For different intervention groups, the first two cans explained 85.34% of the total variance, providing most of the odor information of fecal samples. CDA could differentiate three intervention doses according to the odor information, and the GWP-H group was effectively distinguished from the C group, while samples of the GWP-L and GWP-M group overlapped with the C groups.

With CDA analysis, the effect of different interventions on aging mice could be differentiated with odor information from fecal samples.

### 3.4. Multilayer Perceptron Neural Network (MLP) Results

In this study, the forward structured artificial neural network, a multilayer perceptron neural network (MLP) with multiple node layers, which are fully connected to the next layer, was used. In each layer, there are some neurons related to weighted connections, and the number of these neurons depends on the number of input and output variables of the model [30]. To achieve quantitative discrimination of the intervention process and intervention effect of goat whey powder, MLP neural network analysis was performed on the response signal of the E-nose.

In the discrimination of intervention effects, the responses of 10 E-nose sensors are used as the input layer, and 6 types of intervention (NS, C, Vc, GWP-L, GWP-M, GWP-H) are used as the output layer. A 10-8-6 grid structure is adopted, with 10 neurons in the input layer, 8 neurons in the hidden layer, and 6 neurons in the output layer, and the results are shown in Table 3.

Most of the samples were recognized in the confusion matrix, with the correct classification rate of 66.30%. The basic indicators of the 10-8-6 grid confusion matrix are shown in Table 4. The accuracy, precision, sensitivity, specificity, and specificity area under the curve (AUC) of the intervention effects are 88.75%, 67.25%, 66.25%, 93.25%, and 91.77%, respectively. In summary, the accuracy of the MLP neural network analysis in determining the intervention effect of goat whey powder is above 81.67%. Combined with the MLP neural network, E-nose signals can achieve quantitative differentiation of the intervention effect of goat whey powder.

### 3.5. Rapid Characterization of Antioxidant Indicators for Aging Mice with Different Interventions

To establish a predictive model for antioxidant indicators for aging mice with goat whey powder intervention, the analytical methods of MLR and MLP were used. A dataset containing fecal samples of aging mice with different interventions (184 for calibration and 56 for validation) was used to build the predictive model. The correlation coefficient (R^2^) and root mean square error (RMSE) between predicted and experimental values (RMSE) were used to evaluate the model performance. Larger R^2^ and lower RMSE lead to a good calibration model.

**Figure 8:**
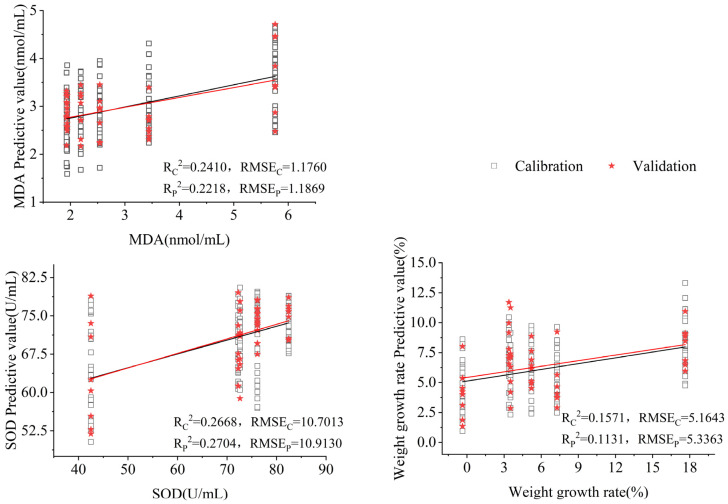
Prediction of antioxidant indicators for aging mice with different interventions by MLR.

The MLR algorithm established a model that describes the relationship between the E-nose sensor signals and antioxidant indicators for aging mice intervention. The predictive models for antioxidant indicators for aging mice with goat whey powder intervention are given in Figure 8.

As shown in Figure 8, the predictive ability of the E-nose was listed. Linear correlation was observed between the responses of E-nose sensors and weight gain rate, SOD, and MDA, with an R^2^ of 0.1571, 0.2668, and 0.2410, respectively.

When the model was used to predict the physiological indexes of aging mice with goat whey protein intervention in the test dataset, R^2^ was higher than 0.1131, thereby showing the possibility of predicting the weight gain rate of aging mice with goat whey protein intervention.

The predictive models built by MLP were effective in predicting antioxidant indices. The coefficients of determination were higher than 0.54, 0.64, and 0.55 for SOD, MDA, and weight-gain rate, respectively. Better predictive results were obtained by MLP. The predictive models for each index are listed in Table 5.

## 4. Discussion

Dietary intake directly affects the composition and metabolic activity of the gut microbiota, which in turn affects the odor of feces [31]. Arising from a complex process involving various bacteria and their metabolites [32], fecal odors can be used as an external indicator of changes in the gut microbiota. The changes in fecal odor were detected by the E-nose sensors. In this study, we found that supplementation with low and medium doses of goat whey protein had a similar effect on fecal odor recovery with that of the Vc group or was even better. This result was consistent with the findings of Long [28], Li [25], El Manouni [21], and Chavez [33], indicating that diet has an effect on fecal odor, and the odor information of the E-nose differed with intervention groups and intervention time.

Fecal odor provides odor information of volatile metabolites of gut microbiota and is used as a supplement for existing methods of evaluating food efficacy and dietary interventions [22,24], as significant correlations were found between fecal odor information and parameters such as SOD, MDA, weight growth rate, and thymus index [28]. Consistent results were found in this research; that is, weight gain rate, MDA content, serum SOD activity, immune indexes of IL-2 and IL-6, and dominant intestinal bacteria are significantly correlated with fecal odor information. The correlation between antioxidant indexes, immune indexes, gut microbiota, and fecal odor is of great significance in reflecting intestinal health status, assisting in the evaluation of the antioxidant ability of goat whey powder.

The changes in volatile organic compounds in the metabolite odor information of exhaled breath, urine, feces, sweat, and blood are used in nutritional intervention and disease diagnosis. Different intervention substances were effectively differentiated between patients with illnesses and healthy controls. The effect of litchi shell proanthocyanidins oligomer [25], dietary cinnamon [26] in vivo, and the difference between breast milk (BM) and formula (FM) feeding [21] were distinguished using fecal odor. In this study, the fecal odor of aging mice varied with different intervention group and time. Combined with CDA, aging mice with different interventions and times could be differentiated using fecal odor information, and MLR and MLP were proven effective predictions of weight gain rate, SOD, and MDA. These results were similar to the intervention volatile organic assessment of bovine whey powder, in which the aging status of mice was assessed by fecal odor response using the E-nose [21], and the dietary supplemental methionine sources significantly influenced odor volatiles in broiler excreta [33]. The MLP neural network analysis results listed all the misclassified samples, and the accuracy of determining the intervention effect of goat whey powder is above 81.67%.

However, few studies have been conducted on the prediction of physiological and biochemical parameters. Based on fecal odor information, it is possible to predict microbial enterotypes using two separate sensors based on a decision-tree model [24]. As reported, the total cholesterol level was predicted by the odor of exhaled air obtained by the E-nose [34]. In our previous study, fecal odor information obtained by the E-nose was used to predict blood pressure [27]. In this study, the antioxidant indexes of SOD, MDA, and weight growth rate were predicted using fecal odor information, although the R^2^ needs to be improved. The predictive model built by MLP was better than that of the MLR, which is easy to use and has relatively good results [34].

In this study, we established a fast and non-invasive method to track changing trends and evaluate the effect of dietary intervention using the odor of feces. A strong correlation was observed between the E-nose response and biochemical indexes of aging mice with bovine whey protein intervention. And with MLR and MLP, the weight gain rate, MDA, and SOD for aging mice were predicted. Therefore, non-invasive detection of fecal odor can serve as a supplementary method for the early-stage evaluation of the effects of food, active ingredients, and pharmaceutical ingredients on animals, as well as for long-term effect tracking. This method offers the advantages of being fast, occurring in real time, and being low-cost. With the development of portable devices, non-invasive monitoring of fecal odor might become an important tool for real-time, rapid, and long-term monitoring of intestinal health in daily home health management.

## 5. Conclusions

Compared with the invasive detection of antioxidant ability and serum antibodies and cytokines, tracking the goat whey protein effect on aging mice based on odor information of volatile metabolites in feces using an E-nose is a simple and fast process. In the future, by utilizing modern machine learning techniques, including more samples and animal models, fecal odor-based rapid and non-invasive detection methods could be used to track changing trends and evaluate the effect of dietary intervention.

## Figures and Tables

**Table 1:** Sensors used and their main applications with the PEN3 electronic nose.

Short Name	Sensor Name	General Description	Reference
S1	W1C-aromatic	Aromatic compounds	Toluene, 10 ppm
S2	W5S-broadrange	Very sensitive, broad range sensitivity, reacts with nitrogen oxides, very sensitive with negative signal	NO_2_, 1 ppm
S3	W3C-aromatic	Ammonia, used as sensor for aromatic compounds	Benzene, 1 ppm
S4	W6S-hydrogen	Mainly hydrogen, selectively (breath gases)	H_2_, 100 ppb
S5	W5C-arom-aliph	Alkanes, aromatic compounds, less polar compounds	Propane, 1 ppm
S6	W1S-broad-methane	Sensitive to methane (environment) ca, 10 ppm, broad range, similar to No. 8	CH_4_, 100 ppm
S7	W1W-sulphur-oganic	Reacts with sulfur compounds, H_2_S 0.1 ppm. Otherwise, sensitive to many terpenes and sulfur organic compounds, which are important for smell, limonene, pyrazine	H_2_S, 1 ppm
S8	W2S-broad-alcohol	Detects alcohols, partially aromatic compounds, broad range	CO, 100 ppm
S9	W2W-sulph-chlor	Aromatic compounds, sulfur organic compounds	H_2_S, 1 ppm
S10	W3S methane-aliph	Reacts with high concentrations > 100 ppm, sometimes very selective (methane)	CH_4_, 100 ppm

**Table 2:** Results of Ducan’s test for the sensor responses of fecal samples after intervention.

Intervention Groups	Sensor Response Value (G/G_0_)									
	S1	S2	S3	S4	S5	S6	S7	S8	S9	S10
C	0.887 ^a^	1.685 ^a^	0.944 ^a^	1.186 ^a^	1.003 ^ab^	2.885 ^bc^	5.241 ^a^	1.667 ^d^	1.714 ^a^	1.277 ^d^
NS	0.816 ^c^	1.157 ^c^	0.912 ^c^	1.151 ^bc^	0.999 ^b^	3.462 ^b^	3.299 ^c^	2.069 ^b^	1.567 ^b^	1.461 ^b^
Vc	0.872 ^ab^	1.434 ^b^	0.937 ^a^	1.156 ^bc^	1.007 ^ab^	2.959 ^c^	4.105 ^b^	1.744 ^cd^	1.562 ^b^	1.317 ^cd^
GWP-L	0.876 ^a^	1.501 ^b^	0.940 ^ab^	1.186 ^a^	1.008 ^a^	2.879 ^c^	4.674 ^ab^	1.728 ^cd^	1.633 ^ab^	1.318 ^cd^
GWP-M	0.852 ^b^	1.192 ^c^	0.930 ^b^	1.150 ^c^	1.004 ^ab^	3.181 ^bc^	4.332 ^b^	1.852 ^c^	1.622 ^ab^	1.356 ^c^
GWP-H	0.742 ^d^	0.932 ^d^	0.864 ^d^	1.169 ^b^	1.003 ^ab^	4.996 ^a^	3.399 ^c^	2.643 ^a^	1.654 ^ab^	1.644 ^a^

Note: Means within the same column followed by the different small letters are significantly different at *p* < 0.05.

**Table 3:** Results of the intervention effect of the discrimination confusion matrix.

Types	Samples	C	Ns	Vc	GWP-L	GWP-M	GWP-H
Effect evaluation	C	25	5	6	1	3	0
	Ns	1	23	3	12	1	0
	Vc	5	8	20	4	2	1
	GWP-L	4	13	3	20	0	0
	GWP-M	5	1	3	0	31	0
	GWP-H	0	0	0	0	0	40
Correct classification rate	66.30%						

**Table 4:** Performance parameters of 10-6-6 grid MLP classifier.

Types	Samples	Accuracy/%	Precision/%	Sensitivity/%	Specificity/%	AUC/%
Intervention groups	C	87.50 (83.32, 91.68)	62.50	62.50	92.50	90.80
	Ns	81.67 (76.79, 86.55)	46.00	57.50	86.50	100
	Vc	85.42 (80.95, 89.89)	57.14	50.00	92.50	87.60
	GWP-L	84.58 (79.93, 89.23)	54.05	50.00	91.50	97.70
	GWP-M	93.75 (90.69, 96.81)	83.78	77.50	97.00	88.10
	GWP-H	99.58 (98.78, 100.38)	100	100	99.50	86.40
	Average	88.75 (85.08, 92.42)	67.25	66.25	93.25	91.77

**Table 5:** Prediction of antioxidant indexes based on MLP.

Prediction Methods	Antioxidant Index	Calibration		Validation	
		R^2^	RMESC	R^2^	RMESP
MLP	SOD	0.5352	0.5376	0.5092	0.5196
	MDA	0.6361	0.638	0.6242	0.6322
	Weight gain rate	0.5475	0.5499	0.5257	0.5358

## Data Availability

Data are contained within the article.
